# Rapid HIV Testing Is Highly Acceptable and Preferred among High-Risk Gay And Bisexual Men after Implementation in Sydney Sexual Health Clinics

**DOI:** 10.1371/journal.pone.0123814

**Published:** 2015-04-21

**Authors:** Damian P. Conway, Rebecca Guy, Stephen C Davies, Deborah L. Couldwell, Anna McNulty, Don E. Smith, Phillip Keen, Philip Cunningham, Martin Holt, Sydney Rapid HIV Test Study

**Affiliations:** 1 The Kirby Institute, University of New South Wales, Sydney, New South Wales, Australia; 2 Short Street Sexual Health Centre, St George Hospital, Kogarah, New South Wales, Australia; 3 North Shore Sexual Health Service, Royal North Shore Hospital, St Leonards, New South Wales, Australia; 4 Sydney Medical School, University of Sydney, Sydney, New South Wales, Australia; 5 Western Sydney Sexual Health Centre, Western Sydney Local Health District, Sydney, New South Wales, Australia; 6 The Marie Bashir Institute for Infectious Diseases and Biosecurity, University of Sydney, Sydney, New South Wales, Australia; 7 Sydney Sexual Health Centre, Sydney Hospital, Sydney, New South Wales, Australia; 8 School of Public Health and Community Medicine, University of New South Wales, Sydney, New South Wales, Australia; 9 Albion Centre, Surry Hills, New South Wales, Australia; 10 St Vincent’s Centre for Applied Medical Research, University of New South Wales, Sydney, New South Wales, Australia; 11 NSW State Reference Laboratory for HIV, St Vincent’s Hospital, Darlinghurst, New South Wales, Australia; 12 Centre for Social Research in Health, University of New South Wales, Sydney, New South Wales, Australia; David Geffen School of Medicine at UCLA, UNITED STATES

## Abstract

**Background:**

Rapid HIV testing (RHT) is well established in many countries, but it is new in Australia. We assessed the acceptability of RHT and its associations among gay, bisexual and other men who have sex with men (GBM) after implementation of RHT in Sydney sexual health clinics.

**Methods:**

GBM were invited to complete an acceptability questionnaire before and after provision of the result of finger-prick blood RHT, comparing their experience of RHT with conventional HIV testing (CHT) involving venipuncture. Logistic regression was used to assess associations between patient characteristics and the preference for RHT over CHT next time they tested for HIV.

**Results:**

Of 1061 GBM who received non-reactive RHT results, 59% found RHT less stressful than CHT and 34% reported no difference, and 61% found RHT more comfortable than CHT and 26% reported no difference. Nearly all men were satisfied with RHT result delivery (99%) and the RHT process overall (99%). Most men (79%) preferred RHT for their next HIV test and this preference was stronger in men who were aged 35-44 years (adjusted odds ratio [AOR] 2.49, p<0.01), reported they would test more often if RHT was available (AOR 1.66, p=0.01), found returning for results annoying (AOR 1.67, p=0.01), and found RHT less stressful (AOR 2.37, p<0.01) and more comfortable (AOR 1.62, p=0.02) than CHT. Men concerned about the reliability of RHT were less than half as likely to prefer RHT for their next HIV test (AOR 0.44, p<0.01).

**Conclusions:**

Most GBM preferred RHT to CHT next time and this preference was associated with finding RHT more convenient, more comfortable and less stressful than CHT. These findings suggest that in a clinic setting RHT should be considered to improve the patient experience and may potentially increase uptake and frequency of HIV testing.

## Introduction

HIV infection remains a major public health issue worldwide, with gay, bisexual and other men who have sex with men (GBM) disproportionately affected [1]. Globally, it is estimated 35 million people are living with HIV infection and there were 1.6 million AIDS-related deaths in 2012 [2]. In recent years, risk behaviours and the number of new HIV diagnoses reported among GBM have been increasing [3–7]. Clinical guidelines recommend at least annual HIV testing for all sexually active GBM and more frequent testing 3–6 monthly for higher risk men [8,9]. However, it is estimated that 10–20% of Australian HIV-infected GBM are unaware of their status and men are not testing for HIV as frequently as recommended [10–12].

HIV testing is a key prevention strategy and earlier identification of HIV infection through more frequent testing and timely initiation of antiretroviral therapy may have both individual and wider community benefits [13–16]. While there is a need for increased uptake and frequency of HIV testing among GBM, testing at the recommended frequency for men at high risk of HIV may involve many clinic attendances and interactions [17,18]. Conventional HIV testing (CHT) involves venipuncture blood specimen collection at a clinic, laboratory-based testing and result delivery (typically one week later) during a second clinic visit or telephone consultation [19]. Commonly reported barriers to conventional testing among GBM include inconvenience, finding the time to test and having to return for results [19,20]. Rapid HIV testing (RHT) at the point-of-care typically involves finger-prick blood or oral fluid specimen collection with results provided during the same visit. For the great majority with non-reactive RHT results, this may make testing more convenient. Adopting RHT may therefore reduce barriers to and increase the acceptability of testing, facilitating more frequent testing in high-risk populations [21,22].

Evaluations of RHT have demonstrated high acceptability among GBM and other patients, with RHT preferred to CHT [23–25]. RHT has successfully reached high-risk populations in community and clinical settings, including patients never previously tested [26,27]. Patients prefer receiving their results quickly and would recommend RHT to their peers [24,27]. However, some patients express concern regarding the reliability of RHT and their clinic visit being prolonged [28]. Since these evaluations of RHT acceptability were conducted using HIV antibody only rapid tests, fourth generation rapid testing that incorporates both HIV antibody and antigen components has become available. The Determine HIV Combo (Alere, Chiba, Japan) was the first-ever fourth generation rapid test and it was licensed for point-of-care use in Australia in 2012, following government policy changes in 2011. One Australian study has found that GBM prefer RHT to CHT and find waiting one week for test results anxiety-provoking [29]. However, there are no local published data on the characteristics of clinic-based GBM who prefer RHT to CHT or the acceptability of different components of the RHT process (which may affect repeat testing). Understanding these elements is important as RHT availability and uptake increase across Australia.

We assessed the acceptability of the RHT process among GBM patients and factors associated with preferring RHT over CHT after implementation of RHT using the Alere Determine HIV Combo assay in Sydney sexual health clinics.

## Methods

### Setting

The Sydney Rapid HIV Test Study was conducted in four free-to-access publicly funded sexual health clinics with high caseloads of GBM: two in central and two in suburban Sydney. The study aimed to assess rapid test performance, the acceptability of RHT to patients and providers and the barriers to HIV testing among GBM. Rapid test performance was compared with the standard of care laboratory serology assays used in this setting and barriers to testing and patient and staff acceptability were assessed via surveys. The performance and provider acceptability data and methodology have previously been published [30,31]. Among GBM surveyed in New South Wales in 2013, 45% of men who had ever tested for HIV reported that their most recent test was at a public sexual health clinic [32].

### Ethical statement

The study was approved by the Human Research Ethics Committees of St Vincent’s Hospital, Darlinghurst and University of New South Wales (UNSW), Sydney. Clinicians obtained informed written consent from patients prior to RHT.

### Study population

Men were eligible for RHT if they were aged 18 years or more, reported sexual contact with another man and requested HIV testing during their visit. GBM were the target population for the study as they account for 85% of new HIV diagnoses in Australia and are the local population with highest HIV prevalence and risk of incident infection [4,33]. Patients known to be HIV-infected were excluded.

### Recruitment

GBM were identified during triage and offered RHT during consultations if they requested HIV testing. The number of eligible GBM that declined RHT or were not enrolled (as they were not offered RHT) was recorded and these patients received CHT.

### Staff training and quality management

Clinic staff completed training on study procedures and RHT using the Determine HIV Combo assay prior to enrolling patients in the study [30]. Site visits were conducted during the course of the study, there was internal quality control testing at the sites each week and RHT results were tracked at each site via the site coordinators. The site investigators and site coordinators supervised and provided support to clinic staff during the course of the study to ensure study procedures were observed.

### Process for rapid testing

Pre-test discussion included the limitations of RHT; including the longer window period (compared with the laboratory fourth generation HIV immunoassays that were standard of care at the clinic sites) and the possibility of false test results. Patients were advised that all RHT results required confirmation by conventional laboratory serology as the rapid test used during the survey period was not yet licensed by the Therapeutic Goods Administration (it was licensed in December 2012). Venipuncture was also necessary as the GBM participants required serology testing for syphilis (and viral hepatitis where appropriate). Clinicians collected finger-prick specimens for RHT and venipuncture blood specimens for serology; swabs and urine specimens for sexually transmitted infection (STI) testing were collected either by the patient or the clinician. Finger-prick blood specimens were applied to the Determine HIV Combo assay and results were read after 20 minutes as per manufacturer package insert instructions [34]. The test kit was moved out of sight of the patient during incubation. Patients received their RHT results during their visit from the clinician (with counselling support provided for patients receiving a reactive result) and they received their CHT and STI testing results (typically one week later) either by telephone or in person at follow-up visits.

### Patient acceptability survey

From October 2011 to August 2012, all GBM participants were invited to self-complete acceptability questionnaires during the clinic visits where they received RHT. Men could participate in the survey once only and no promotion of RHT occurred during the survey period, either externally to the GBM community or internally within the clinic sites. The survey was focus tested among GBM patients during its development. Some questions were adapted from behavioural surveillance questionnaires [35] and questions regarding patient attitudes and satisfaction used five-point Likert scales (with responses ranging from strongly agree to strongly disagree) (S1 Questionnaire). Part 1 assessed patient demographics, sexual risk behaviour, previous HIV testing patterns, reasons for and barriers to HIV testing, and patient perception of the reliability of RHT. Part 2 of the survey was completed after patients received their rapid test result, but was not completed if the result was reactive as the patient’s well-being took priority. Part 2 assessed patient experience of RHT in terms of the duration of their clinic visit, the level of stress and anxiety, and the comfort of specimen collection; comparing that with their experience of CHT (in those men that had tested for HIV previously). It also assessed their satisfaction with components of the RHT process, their willingness to pay for RHT and whether they would prefer RHT over CHT next time they tested for HIV (if RHT was available).

### Statistical analysis

Descriptive statistics were used to report responses given by all participants to part 1 survey questions, excluding patients with missing surveys or missing rapid test results. The age of eligible men who were and were not enrolled was compared with the Mann-Whitney test. Chi-squared tests were used to compare men aged less than 30 years with those aged 30 or more regarding the proportion of men reporting ever testing for HIV and the proportion of men reporting concern about the reliability of RHT. Analyses of part 2 survey questions excluded patients with reactive rapid tests and those who did not complete part 2. Logistic regression was used for bivariate analyses to assess associations between individual patient characteristics (including those that can be used to identify high-risk patient populations) and preferring RHT when testing for HIV next time. On bivariate analysis, if a variable category had a probability value of less than 0.05, that variable was entered into the multivariate analysis (block entry method). Missing data were included to maintain power in the analysis and if the proportion of missing data for variables was greater than 2%, missing data were re-coded into their own category (but not the reference category). P-values of less than 0.05 in the multivariate analysis were considered significant. Analysis was also conducted with missing data excluded and with never testers excluded to check for any variation in the outcomes. Analyses were performed using Stata (Release 12, StataCorp LP, College Station, Texas) (S1 Dataset).

## Results

### Patients that were not enrolled or were excluded from analysis

**Fig 1:**
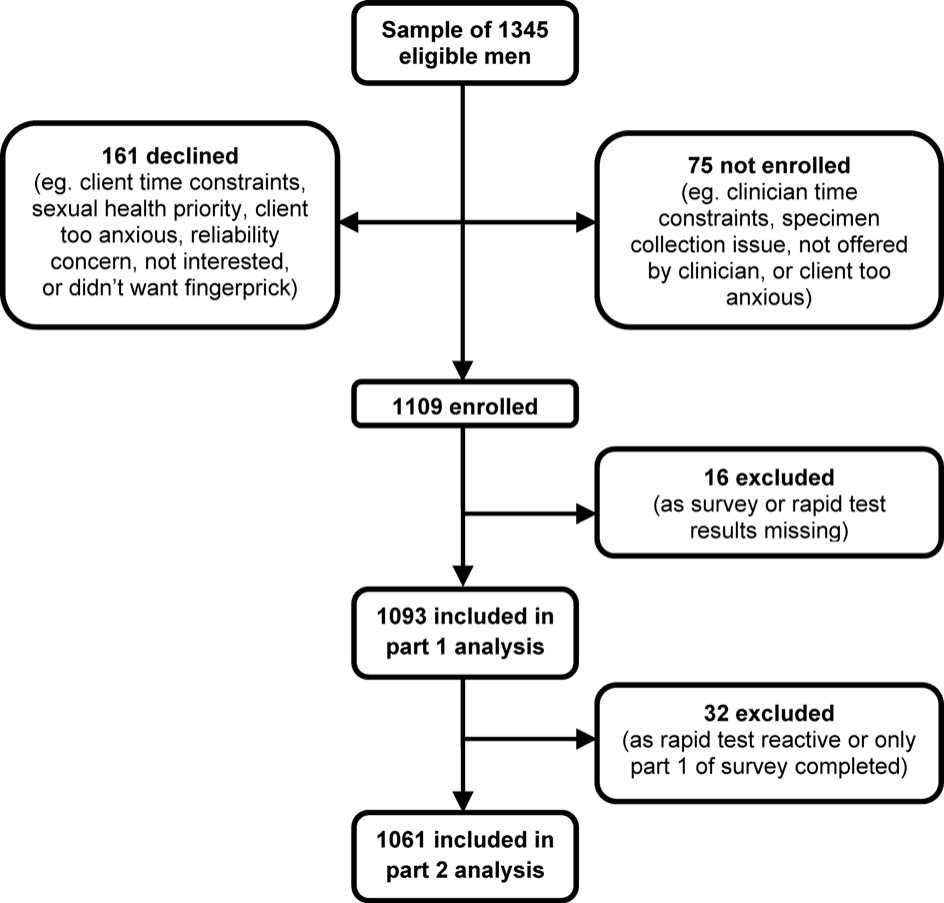
Flowchart of patient participation in the study.

Of 1345 eligible GBM patients at the four sites, 1109 (82.5%) were enrolled, 161 (12.0%) declined, and 75 (5.6%) were not enrolled (Fig 1). Among those for whom a reason was recorded, the main reason for patients declining enrolment was patient time constraints (52.0%) and the main reason for patients not being enrolled was clinician time constraints (62.1%). Of 1109 enrolled patients, 1093 (98.6%) were included in the part 1 survey analysis (16 patients were excluded because their survey or rapid test results were missing) and 1061 (95.7%) were included in the part 2 survey analysis (32 patients were excluded because they had reactive rapid tests or had only completed part 1 of the survey). The median age of enrolled men did not differ from those who declined and/or were not enrolled (30 versus 31 years; p = 0.12).

### Participant characteristics

Of 1093 participants, 87.9% were gay-identified, while 10.7% identified as bisexual and 1.1% as heterosexual. Ever testing for HIV was reported by 89.9% of these men and 67.3% reported testing for HIV in the past year. Men aged 18–29 years were less likely to have ever tested than men aged 30 years or more (85.0% versus 94.1%; χ^2^ = 24.3, p<0.01). Regarding usual testing frequency, 54.6% of men were testing at least twice yearly and 23.9% annually (Table 1). Having sex with casual male partners only in the last 6 months was reported by 36.5% of participants, while 21.1% reported regular male partners only and 32.5% reported both casual and regular male partners. Of those with regular male partners, 32.0% reported their partner’s HIV status was positive or unknown. Of those who reported anal sex with casual male partners, 39.9% reported unprotected anal sex with those partners in the last six months. Having more than 10 male sexual partners in the last six months was reported by 28.3% of participants. Among 234 men who reported testing for HIV less than annually or who had never tested, 17.8% had more than 10 male sexual partners in the last six months and 32.6% reported unprotected anal sex with casual male partners.

**Table 1:** Characteristics of participants (N = 1093).

Characteristic	N^1^ (%)
Sexual identity	
Gay	956 (87.9)
Bisexual/straight/other	132 (12.1)
Age	
18–24 years	218 (20.2)
25–34 years	491 (45.4)
35–44 years	252 (23.3)
More than 45 years	120 (11.1)
Ever tested for HIV	
Yes	981 (89.9)
No/don’t know	110 (10.1)
Frequency of HIV testing	
Four times a year	244 (22.5)
Twice a year	349 (32.1)
Once a year	260 (23.9)
Less than once a year/never tested	234 (21.5)
Concerned about the reliability or accuracy of rapid HIV testing	
Yes	234 (21.5)
No/don’t know	853 (78.5)
Would test more often if rapid HIV testing was available^2^	
Yes	725 (66.5)
No/don’t know	365 (33.5)
Preferred site for rapid HIV testing	
Sexual health clinic	815 (74.8)
Home	137 (12.6)
General practice	61 (5.6)
Other^3^	77 (7.1)
Number of male sex partners in last 6 months	
None/one	131 (12.1)
2–5 men	451 (41.7)
6–10 men	194 (17.9)
More than 10 men	306 (28.3)
Type of male sex partners in last 6 months	
Casual partners only	399 (36.5)
Regular partner(s) only	231 (21.1)
Both casual & regular partners	355 (32.5)
Neither casual nor regular partners	108 (9.9)
HIV status of regular male partner^4^	
Positive	39 (7.2)
Unknown	134 (24.8)
Negative	368 (68.0)
Describe relationship with regular male partner^4^	
Monogamous	195 (33.1)
Open	395 (67.0)
Condom use for anal intercourse with casual male partners in last 6 months^5^	
Never/sometimes	379 (39.9)
Always	570 (60.1)

^1^ Men with missing data excluded

^2^ Before rapid HIV test result was received

^3^ Pharmacy, community organisation or gay venue

^4^ Men with no regular male partner excluded

^5^ Men with no anal intercourse with casual partners and no casual male partners excluded

### Acceptability of rapid testing prior to test result

Prior to receiving RHT results, 66.5% of participants reported they would test more often if RHT was available (Table 1). Preferring sexual health clinic sites for RHT was reported by 74.8%, while 12.6% indicated they would prefer rapid testing at home. Being concerned about the reliability or accuracy of RHT was reported by 21.5% of participants, while 56.9% were not concerned and 21.6% didn’t know. Men aged 18–29 years were more likely to be concerned about the reliability of rapid testing than men aged 30 years or more (27.1% versus 16.9%; χ^2^ = 16.7, p<0.01).

### Acceptability after rapid testing result

Among participants who had previously tested for HIV, 63.1% perceived their RHT clinic visit as being the same length or shorter than their previous visits and 59.4% reported there was less stress and anxiety with RHT (Table 2). When asked to compare the comfort of lancet puncture for finger-prick specimen collection for RHT to venipuncture for CHT, 60.7% of participants found the former more comfortable. There was overwhelming patient satisfaction regarding components of the RHT process and RHT overall: 97.6% were satisfied with pre-test-discussion for RHT, 98.6% with RHT result delivery for 98.6% with RHT overall. Also, 93.4% of patients said they would recommend RHT to others. Preferring RHT to CHT when testing for HIV next time was reported by 78.8% of participants and 50.3% of participants were prepared to pay for RHT (if not government funded).

**Table 2:** Acceptability of rapid HIV testing (after result) (N = 1061).

Characteristic	N^1^ (%)
Duration of clinic visit today versus previous clinic visit^2^	
Shorter	266 (27.2)
About the same	351 (35.9)
Longer	361 (36.9)
Stress and anxiety of rapid HIV testing today versus previous HIV testing^2^	
Less stress and anxiety	587 (59.4)
About the same	331 (33.5)
More stress and anxiety	70 (7.1)
Comfort of finger-prick specimen collection versus venipuncture today	
Less comfortable	130 (13.7)
About the same	243 (25.6)
More comfortable	575 (60.7)
Satisfied with pre-test discussion about rapid HIV testing	
Yes	1035 (97.6)
No	25 (2.4)
Satisfied with rapid HIV testing result delivery	
Yes	1035 (98.6)
No	15 (1.4)
Satisfied with rapid HIV testing overall	
Yes	1037 (98.6)
No	15 (1.4)
Would recommend rapid HIV testing to others	
Yes	983 (93.4)
No	69 (6.6)
Would prefer rapid HIV testing for next HIV test	
Yes	830 (78.8)
No	223 (21.2)
Amount patient would pay for rapid HIV test in private clinic if not government funded	
Nothing, it should be government funded	524 (49.7)
$15–20	334 (31.7)
$30–50	197 (18.7)

^1^ Men with reactive rapid tests and men with missing data excluded.

^2^ Men who had not tested for HIV before excluded.

### Factors associated with preferring rapid testing next time

In bivariate analysis, preferring RHT next time was not associated with the following characteristics that have been used as indicators of higher behavioural risk in GBM populations: the number of male sexual partners reported in the previous six months; reporting unprotected anal intercourse with casual male partners; or reporting a regular male partner of HIV-positive or unknown status. In multivariate analysis, preferring RHT next time was independently associated with being aged 35–44 years (reference category 45 years or more; adjusted odds ratio [AOR] 2.49, p<0.01); reporting you would test more often if RHT was available (AOR 1.66, p = 0.01); finding returning for results annoying (AOR 1.67, p = 0.01); reporting less stress and anxiety with RHT than with previous CHT (AOR 2.37, p<0.01); and finding finger-prick specimen collection more comfortable than venipuncture (AOR 1.62, p = 0.02) (Table 3). However, men who expressed concern about the reliability of RHT were less than half as likely to prefer RHT next time (AOR 0.44, p<0.01). Multivariate analysis conducted with missing data excluded and with never testers excluded produced the same results in terms of these variable categories being independently associated with preferring RHT next time; except that reporting you would test more often if RHT was available (p = 0.08) and finding finger-prick specimen collection more comfortable than venipuncture (p = 0.05) were no longer significant with missing data excluded.

**Table 3:** Variables associated with preference for rapid HIV testing next time^1^ (N = 1061).

Variable	Category	Odds ratio	p-value	Adjusted odds	p-value
		(95% CI)		ratio (95% CI)	
Age	18–24 yrs	0.89 (0.54–1.44)	0.63	0.81 (0.46–1.43)	0.46
	25–34 yrs	1.33 (0.86–2.08)	0.20	1.19 (0.72–1.96)	0.51
	35–44 yrs	2.53 (1.47–4.35)	<0.01	2.49 (1.38–4.49)	**<0.01**
	More than 45 yrs	1.00 ref	-	1.00 ref	-
How long ago patient was last tested for HIV	6 months or less	2.05 (1.21–3.47)	0.01	1.24 (0.40–3.80)	0.71
	7–12 months	1.74 (0.95–3.20)	0.07	0.90 (0.28–2.93)	0.86
	1–2 yrs	1.91 (1.05–3.47)	0.03	1.04 (0.33–3.30)	0.94
	More than 2 yrs	1.72 (0.87–3.38)	0.12	0.89 (0.29–2.67)	0.83
	No prior test	1.00 ref	-	1.00 ref	-
HIV status of regular male partner	Positive	0.84 (0.39–1.79)	0.65	0.77 (0.31–1.92)	0.57
	Don’t know	1.44 (0.89–2.34)	0.14	1.77 (0.97–3.25)	0.06
	Negative	1.42 (1.02–1.98)	0.04	1.44 (0.88–2.38)	0.15
	No regular partner	1.00 ref	-	1.00 ref	-
Concerned about the accuracy or reliability of rapid HIV testing	Yes	0.44 (0.31–0.62)	<0.01	0.44 (0.30–0.65)	**<0.01**
	No	1.00 ref	-	1.00 ref	-
	Don’t know	0.59 (0.41–0.85)	<0.01	0.67 (0.45–1.00)	0.05
Would test more often if rapid HIV testing was available	Yes	1.97 (1.39–2.80)	<0.01	1.66 (1.11–2.49)	**0.01**
	No	1.00 ref	-	1.00 ref	-
	Don’t know	0.67 (0.43–1.06)	0.09	0.74 (0.45–1.24)	0.25
Prefers rapid HIV testing outside sexual health clinic	Yes	1.66 (1.15–2.40)	0.01	1.35 (0.90–2.03)	0.15
	No	1.00 ref	-	1.00 ref	-
Finds it difficult to get an appointment for HIV test	Yes	4.25 (1.31–13.83)	0.02	2.15 (0.59–7.77)	0.24
	No	1.00 ref	-	1.00 ref	-
Dislikes needles	Yes	2.14 (1.15–3.98)	0.02	1.93 (0.98–3.82)	0.06
	No	1.00 ref	-	1.00 ref	-
Finds it stressful waiting for HIV test results	Yes	1.53 (1.08–2.16)	0.02	1.17 (0.79–1.74)	0.43
	No	1.00 ref	-	1.00 ref	-
Finds it annoying to have to return for HIV results	Yes	1.92 (1.35–2.72)	<0.01	1.67 (1.12–2.49)	**0.01**
	No	1.00 ref	-	1.00 ref	-
Currently has sex with regular male partner(s)	Yes	1.41 (1.05–1.88)	0.02	1.07 (0.51–2.23)	0.86
	No	1.00 ref	-	1.00 ref	-
Description of relationship with regular male partner	Monogamous	1.05 (0.70–1.56)	0.83	0.80 (0.35–1.85)	0.61
	Open	1.46 (1.04–2.05)	0.03	0.92 (0.42–2.02)	0.84
	No regular partner	1.00 ref	-	1.00 ref	-
	Missing data	0.83 (0.43–1.60)	0.58	0.79 (0.38–1.66)	0.54
Compared with last HIV test, duration of clinic visit today	Shorter	1.50 (0.97–2.30)	0.07	1.15 (0.72–1.85)	0.57
	About the same	1.00 ref	-	1.00 ref	-
	Longer	0.70 (0.50–1.00)	0.05	0.83 (0.56–1.23)	0.36
	No prior test	0.47 (0.28–0.80)	0.01	0.43 (0.13–1.42)	0.17
Compared with last HIV test, amount of stress and anxiety experienced today	Less stress	2.94 (2.12–4.08)	<0.01	2.37 (1.63–3.45)	**<0.01**
	About the same	1.00 ref	-	1.00 ref	-
	More stress	1.36 (0.78–2.38)	0.28	1.57 (0.85–2.91)	0.15
	No prior test	0.96 (0.55–1.68)	0.87	2.44 (0.74–8.01)	0.14
Compared with venipuncture, comfort of finger-prick today	Less comfort	0.70 (0.44–1.12)	0.14	0.61 (0.36–1.03)	0.06
	About the same	1.00 ref	-	1.00 ref	-
	More comfort	1.78 (1.24–2.57)	<0.01	1.62 (1.08–2.43)	**0.02**
	Missing data	0.69 (0.42–1.12)	0.13	0.61 (0.36–1.03)	0.07
Prepared to pay for rapid HIV testing	Yes	1.45 (1.08–1.94)	0.01	1.33 (0.96–1.84)	0.09
	No	1.00 ref	-	1.00 ref	-

^1^ Men with missing data included^;^ CI = confidence interval; p = probability; ref = reference category

## Discussion

We have found a high degree of acceptance of and satisfaction with the components of the RHT process and with RHT overall among GBM patients after implementation of RHT in public sexual health clinics in Sydney. Most men found RHT less stressful and more comfortable than CHT and nearly all men were satisfied with pre-test discussion, result delivery and the RHT process overall. Most men indicated they would prefer RHT over CHT for their next HIV test and this preference was stronger in men who found RHT more convenient, more comfortable and less stressful than CHT. Men were less likely to prefer RHT next time if they were concerned about the reliability and accuracy of RHT. The greater preference for RHT next time in men aged 35–44 years may be related to their greater testing experience and lesser concern about the reliability of RHT compared with younger men.

The fourth generation Determine HIV Combo rapid test used in our study was designed to increase sensitivity in acute HIV infection by detecting HIV antigen. Though evaluations using fingerprick blood specimens at the point-of-care have not reproduced the sensitivity reported for the assay’s antigen component in laboratory studies, patient participants would not have been aware of the performance data for the assay in our study as it was not reported until after the patient acceptability survey was completed [30,36–38]. The high satisfaction with and strong preference for RHT next time suggests RHT makes the testing process more convenient and may encourage greater uptake of HIV testing and repeat testing. In online and community-based surveys, GBM report that they would test more frequently for HIV if testing was more convenient and there was rapid provision of results [22,39]. Though a recent randomised controlled trial of RHT in clinic-based GBM showed that among those randomised to RHT there was greater initial uptake of HIV testing compared with the conventional testing arm, this increase was not sustained over the two-year trial period [29]. Whether the provision of RHT delivers sustained increases in testing frequency among Australian GBM may require further research involving a range of settings and testing modalities.

Our findings also suggest there are aspects of the process that could be improved to increase acceptability. Over a third (37%) of participants found their clinic visit had been prolonged by RHT. Our study involved written consent, completion of a survey and it used a 20-minute incubation rapid test with the patient receiving their result during their clinic visit. Previous studies have shown patients prefer quick delivery of HIV results within an hour [22,24]; hence, tests with shorter incubation times of 10 minutes or less may be important to consider in future RHT programs and may allow easier integration of RHT into screening consultations [31]. Though other studies have also reported patient concern about the reliability of RHT and that patient preference for RHT was conditional on its performance being similar to that of CHT [28,40], these studies did not assess how that influenced the preference for RHT next time via multivariate analysis.

Our study sample reported high levels of risk behaviours for HIV acquisition, such as high numbers of male sexual partners, having a regular male partner of positive or unknown HIV-status, and having anal sex without condoms with casual male partners. While it is encouraging that half of participants reported testing twice yearly or more often, it is a concern that a fifth were either testing less than annually or had never tested. Some of these infrequent and never testers reported high-risk behaviour, which has also been demonstrated among GBM in primary care [10]. Testing models incorporating RHT may be attractive to GBM not previously tested for HIV given commonly reported barriers to testing in these men such as inconvenience and not having time to test [19,20]. RHT and home self-testing may be adjuncts to CHT for some GBM, who may test more frequently for HIV if they can access testing in settings and in ways that suit them [18,22,41,42]. Home self-testing is now legal in Australia, but as the Therapeutic Goods Administration has not yet licensed a rapid test for home use, some GBM are accessing home testing by purchasing testing kits online [43].

The main strengths of our study were the use of a fourth generation rapid HIV test in a large sample of high-risk GBM, the high participation rate, and the survey which assessed an extended range of patient characteristics and components of the testing process. Our study reflected real world implementation of RHT in a clinical setting and the procedures used at the sites for risk assessment and screening among GBM in the study were those that already existed, except for the addition of a brief consent for rapid testing. All staff involved received the same training regarding study procedures and performance of RHT. The procedure for performing RHT in this study was that recommended by the manufacturer, so we would not expect that to differ from how it is conducted in other settings.

Our evaluation also had some potential limitations. Our findings in clinic-based men may not be generalisable to all GBM, as online surveys have shown higher preferences for home-based rapid testing than that reported in our study [18,41]. We conducted RHT using the Determine HIV Combo assay, but acceptability may vary if a shorter incubation test or an oral fluid test were used. Though our questionnaire was not previously validated, it was developed with expert input from social science researchers and was focus tested with GBM at the clinic sites prior to the study. Our study assessed patient acceptability of RHT in the context of a research study, while acceptability in day-to-day clinical practice may be greater in the absence of study activities and documentation. Though they represent a small proportion of eligible men, the views of those who declined RHT or were not enrolled by clinic staff on RHT acceptability are unknown (as is the effect their views would have on the outcomes). Also, patient satisfaction in our study may have been influenced by having just received a non-reactive test result. We did not assess post-result acceptability in men with reactive tests as providing them with support was our priority, but when acceptability has been assessed in patients with reactive tests they report satisfaction with their choice of test and they would choose the same test again [25,26].

## Conclusion

We have shown high acceptance of and satisfaction with RHT among high-risk GBM patients after its implementation in Sydney sexual health clinics. Prior to their RHT result, two thirds of men reported they would test more often if RHT was available. Post-test result, satisfaction with the process of clinic-based RHT was overwhelming and most men preferred RHT to CHT for their next HIV test. These findings may inform planning for further roll-out of RHT in clinical services to facilitate more frequent testing and earlier HIV diagnosis among GBM patients.

## Supporting Information

S1 Dataset (XLS)

S1 Questionnaire (PDF)

## References

[1] Beyrer C, Baral SD, van Griensven F, Goodreau SM, Chariyalertsak S, Wirtz AL, et al (2012) Global epidemiology of HIV infection in men who have sex with men. The Lancet 380: 367–377. 10.1016/S0140-6736(12)60821-6 PMC3805037 22819660

[2] Maartens G, Celum C, Lewin SR (2014) HIV infection: epidemiology, pathogenesis, treatment, and prevention. The Lancet 384: 258–271. 10.1016/S0140-6736(14)60164-1 24907868

[3] Centers for Disease Control and Prevention (February 2013) HIV Surveillance Report, 2011; Vol. 23. Atlanta, Georgia: U.S. Department of Health and Human Services. Available: http://www.cdc.gov/hiv/pdf/statistics_2011_HIV_Surveillance_Report_vol_23.pdf.

[4] The Kirby Institute (2014) HIV, viral hepatitis and sexually transmissible infections in Australia: Annual Surveillance Report 2014. Sydney: The University of New South Wales Available: https://kirby.unsw.edu.au/sites/default/files/hiv/resources/ASR2014.pdf.

[5] European Centre for Disease Prevention and Control/WHO Regional Office for Europe (2013) HIV/AIDS surveillance in Europe 2012. Stockholm: European Centre for Disease Prevention and Control. Available: http://www.ecdc.europa.eu/en/publications/publications/20121130-annual-hiv-surveillance-report.pdf.

[6] Paz-Bailey G, Hall H, Wolitski R, Prejean J, Van Handel M, Le B, et al (2013) HIV testing and risk behaviors among gay, bisexual, and other men who have sex with men—United States. MMWR Morb Mortal Wkly Rep 62: 958–962. 24280915 PMC4585635

[7] de Wit J, Mao L, Adam P, Treloar CE (2014) HIV/AIDS, hepatitis and sexually transmissible infections in Australia: Annual report of trends in behaviour 2014. Sydney: Centre for Social Research in Health, The University of New South Wales. Available at: https://csrh.arts.unsw.edu.au/research/publications/reports-trends-in-behavior/

[8] Templeton DJ, Read P, Varma R, Bourne C (2014) Australian sexually transmissible infection and HIV testing guidelines for asymptomatic men who have sex with men 2014: a review of the evidence. Sexual Health 11: 217–229. 10.1071/SH14003 24690473

[9] Workowski KA, Berman S (2010) Sexually transmitted diseases treatment guidelines, 2010. MMWR Recomm Rep 59: 1–110. 16888612

[10] Guy R, Goller JL, Spelman T, El-Hayek C, Gold J, Lim M, et al (2010) Does the frequency of HIV and STI testing among MSM in primary care adhere with Australian guidelines? Sexually Transmitted Infections 86: 371–376. 10.1136/sti.2009.040972 20460263

[11] Mallitt K-A, Wilson DP, McDonald A, Wand H (2012) HIV incidence trends vary between jurisdictions in Australia: an extended back-projection analysis of men who have sex with men. Sexual Health 9: 138–143. 10.1071/SH10141 22498157

[12] Birrell F, Staunton S, Debattista J, Roudenko N, Rutkin W, Davis C (2010) Pilot of non-invasive (oral fluid) testing for HIV within a community setting. Sexual Health 7: 11–16. 10.1071/SH09029 20152090

[13] The SPARTAC Trial Investigators (2013) Short-Course Antiretroviral Therapy in Primary HIV Infection. New England Journal of Medicine 368: 207–217. 10.1056/NEJMoa1110039 23323897 PMC4131004

[14] Le T, Wright EJ, Smith DM, He W, Catano G, Okulicz JF, et al (2013) Enhanced CD4+ T-Cell Recovery with Earlier HIV-1 Antiretroviral Therapy. New England Journal of Medicine 368: 218–230. 10.1056/NEJMoa1110187 23323898 PMC3657555

[15] Das M, Chu PL, Santos G-M, Scheer S, Vittinghoff E, McFarland W, et al (2010) Decreases in Community Viral Load Are Accompanied by Reductions in New HIV Infections in San Francisco. PLoS ONE 5: e11068 10.1371/journal.pone.0011068 20548786 PMC2883572

[16] Cohen MS, Gay CL (2010) Treatment to Prevent Transmission of HIV-1. Clinical Infectious Diseases 50: S85–S95. 10.1086/651478 20397961 PMC4147719

[17] Wilson D, Hoare A, Regan D, Law M (2009) Importance of promoting HIV testing for preventing secondary transmissions: modelling the Australian HIV epidemic among men who have sex with men. Sexual Health 6: 19–33. 19254488 10.1071/sh08081

[18] Bavinton BR, Brown G, Hurley M, Bradley J, Keen P, Conway DP, et al (2013) Which gay men would increase their frequency of HIV testing with home self-testing? AIDS Behav 17: 2084–2092. 10.1007/s10461-013-0450-z 23525790

[19] Prestage G, Brown G, Keen P (2012) Barriers to HIV testing among Australian gay men. Sexual Health 9: 453–458. 10.1071/SH12033 23380195

[20] MacKellar DA, Hou S-I, Whalen CC, Samuelsen K, Sanchez T, Smith A, et al (2011) Reasons for Not HIV Testing, Testing Intentions, and Potential Use of an Over-the-Counter Rapid HIV Test in an Internet Sample of Men Who Have Sex With Men Who Have Never Tested for HIV. Sexually Transmitted Diseases 38: 419–428. 10.1097/OLQ.0b013e31820369dd 21183863

[21] Spielberg F, Branson BM, Goldbaum GM, Lockhart D, Kurth A, Celum CL, et al (2003) Overcoming Barriers to HIV Testing: Preferences for New Strategies Among Clients of a Needle Exchange, a Sexually Transmitted Disease Clinic, and Sex Venues for Men Who Have Sex with Men. Journal of Acquired Immune Deficiency Syndromes 32: 318–327. 12626893 10.1097/00126334-200303010-00012

[22] Gray RT, Prestage GP, Down I, Ghaus MH, Hoare A, Bradley J, et al (2013) Increased HIV Testing Will Modestly Reduce HIV Incidence among Gay Men in NSW and Would Be Acceptable if HIV Testing Becomes Convenient. PLoS ONE 8: e55449 10.1371/journal.pone.0055449 23457470 PMC3574096

[23] San Antonio-Gaddy M, Richardson-Moore A, Burstein GR, Newman DR, Branson BM, Birkhead GS (2006) Rapid HIV antibody testing in the New York State Anonymous HIV Counseling and Testing Program: experience from the field. Journal of Acquired Immune Deficiency Syndromes 43: 446–450. 16980908 10.1097/01.qai.0000243055.65698.51

[24] Merchant RC, Clark MA, Seage GR 3rd, Mayer KH, Degruttola VG, Becker BM (2009) Emergency department patient perceptions and preferences on opt-in rapid HIV screening program components. AIDS Care 21: 490–500. 10.1080/09540120802270284 19283644 PMC3173939

[25] Champenois K, Le Gall JM, Jacquemin C, Jean S, Martin C, Rios L, et al (2012) ANRS-COM'TEST: description of a community-based HIV testing intervention in non-medical settings for men who have sex with men. BMJ Open 2: e000693 10.1136/bmjopen-2011-000693 22466158 PMC3323802

[26] Guenter D, Greer J, Barbara A, Robinson G, Roberts J, Browne G (2008) Rapid point-of-care HIV testing in community-based anonymous testing program: a valuable alternative to conventional testing. AIDS Patient Care STDS 22: 195–204. 10.1089/apc.2007.0137 18290752

[27] Smith LV, Rudy ET, Javanbakht M, Uniyal A, Sy LS, Horton T, et al (2006) Client satisfaction with rapid HIV testing: comparison between an urban sexually transmitted disease clinic and a community-based testing center. AIDS Patient Care STDS 20: 693–700. 17052139 10.1089/apc.2006.20.693

[28] Hutchinson AB, Corbie-Smith G, Thomas SB, Mohanan S, del Rio C (2004) Understanding the patient's perspective on rapid and routine HIV testing in an inner-city urgent care center. AIDS Educ Prev 16: 101–114. 15134119 10.1521/aeap.16.2.101.29394

[29] Read TR, Hocking JS, Bradshaw CS, Morrow A, Grulich AE, Fairley CK, et al (2013) Provision of rapid HIV tests within a health service and frequency of HIV testing among men who have sex with men: randomised controlled trial. BMJ 347: f5086 10.1136/bmj.f5086 24004988 PMC3762440

[30] Conway DP, Holt M, McNulty A, Couldwell DL, Smith DE, Davies SC, et al (2014) Multi-Centre Evaluation of the Determine HIV Combo Assay when Used for Point of Care Testing in a High Risk Clinic-Based Population. PloS One 9: e94062 10.1371/journal.pone.0094062 24714441 PMC3979750

[31] Conway DP, Guy R, McNulty A, Couldwell DL, Davies SC, Smith DE, et al (2015) Effect of testing experience and profession on provider acceptability of rapid HIV testing after implementation in public sexual health clinics in Sydney. HIV Medicine: 10.1111/hiv.12209 Epub Jan 21, 2015 25604470

[32] New South Wales Centre for Population Health (2013) Where is HIV testing being done? in: HIV in NSW—Data for Performance Monitoring Report (2nd Quarter update). Sydney: New South Wales Ministry of Health. pp. 12. Available at: http://www.health.nsw.gov.au/endinghiv/Documents/hiv-in-nsw-2nd-quarter-report-2013.pdf

[33] Poynten IM, Jin F, Prestage GP, Kaldor JM, Kippax S, Grulich AE (2010) Defining high HIV incidence subgroups of Australian homosexual men: implications for conducting HIV prevention trials in low HIV prevalence settings. HIV Med 11: 635–641. 10.1111/j.1468-1293.2010.00833.x 20456511

[34] Alere Medical Co. Ltd (2011) Determine HIV-1/2 Ag/Ab Combo Rapid Test Package Insert. Alere Medical Co., Ltd, Chiba, Japan.

[35] Hull P, Holt M, Mao L, Kao S-C, Prestage G, Zablotska I, et al. (2011) Sydney Gay Community Periodic Survey Report 2011. Sydney, Australia: National Centre in HIV Social Research, UNSW. Available at: https://csrh.arts.unsw.edu.au/research/publications/hiv-sexual-health/

[36] Beelaert G, Fransen K (2010) Evaluation of a rapid and simple fourth-generation HIV screening assay for qualitative detection of HIV p24 antigen and/or antibodies to HIV-1 and HIV-2. J Virol Methods 168: 218–222. 10.1016/j.jviromet.2010.06.002 20561542

[37] Masciotra S, Luo W, Youngpairoj AS, Kennedy MS, Wells S, Ambrose K, et al (2013) Performance of the Alere Determine HIV-1/2 Ag/Ab Combo Rapid Test with specimens from HIV-1 seroconverters from the US and HIV-2 infected individuals from Ivory Coast. Journal of Clinical Virology 58, Supplement 1: e54–e58. 10.1016/j.jcv.2013.07.002 23911678 PMC11111262

[38] Rosenberg NE, Kamanga G, Phiri S, Nsona D, Pettifor A, Rutstein SE, et al (2012) Detection of Acute HIV Infection: A Field Evaluation of the Determine HIV-1/2 Ag/Ab Combo Test. Journal of Infectious Diseases 205: 528–534. 10.1093/infdis/jir789 22207651 PMC3318673

[39] Chen MY, Bilardi JE, Lee D, Cummings R, Bush M, Fairley CK (2010) Australian men who have sex with men prefer rapid oral HIV testing over conventional blood testing for HIV. Int J STD AIDS 21: 428–430. 10.1258/ijsa.2010.009552 20606224

[40] Debattista J, Bryson G, Roudenko N, Dwyer J, Kelly M, Hogan P, et al (2007) Pilot of non-invasive (oral fluid) testing for HIV within a clinical setting. Sexual Health 4: 105–109. 17524288 10.1071/sh07014

[41] Sharma A, Stephenson RB, White D, Sullivan PS (2014) Acceptability and intended usage preferences for six HIV testing options among internet-using men who have sex with men. Springerplus 3: 109 10.1186/2193-1801-3-109 24600551 PMC3942559

[42] Bilardi JE, Walker S, Read T, Prestage G, Chen MY, Guy R, et al (2013) Gay and bisexual men's views on rapid self-testing for HIV. AIDS Behav 17: 2093–2099. 10.1007/s10461-012-0395-7 23297083

[43] Therapeutic Goods Administration (2014) HIV self-tests in Australia: questions and answers. Australian Government Department of Health. Available: https://www.tga.gov.au/community-qa/hiv-self-tests-australia-questions-and-answers.

