# Supplementary material for: Rapid HIV Testing Is Highly Acceptable and Preferred among High-Risk Gay And Bisexual Men after Implementation in Sydney Sexual Health Clinics
Source: PLoS One. 2015 Apr 21;10(4):e0123814. doi: 10.1371/journal.pone.0123814 (PMC4405382; doi:10.1371/journal.pone.0123814)
Supplement: S1 Questionnaire — (PDF) [file pone.0123814.s002.pdf]

Rapid HIV testing is highly acceptable and preferred among high-risk gay and bisexual men after implementation in Sydney sexual health clinics

PLOS One 2015

Damian P Conway *, Rebecca Guy, Stephen C Davies, Deborah L Couldwell, Anna McNulty, Don E Smith, Phillip Keen, Philip Cunningham, Martin Holt on behalf of the Sydney Rapid HIV Test Study

* Corresponding author

Email: dconway@kirby.unsw.edu.au

Supplemental information file S1

Sydney Rapid HIV Test Study – Patient acceptability questionnaire

PART ONE

1. Do you think of yourself as

   1☐Gay/homosexual 2☐Bisexual 3☐Heterosexual
   4☐Other (please specify) [ ]

2. How old are you?

   ☐☐ Years

3. Have you ever had an HIV test?

   1☐Yes 2☐No 3☐Don’t know/Unsure

4. When did you last test for HIV?

   1☐Less than a week ago 2☐1-4 weeks ago 3☐1–6 months ago 4☐7–12 months ago
   5☐1–2 years ago 6☐2–4 years ago 7☐More than 4 years ago 8☐Never tested

5. If you have a **regular** male partner, do you know the result of his HIV antibody test?

   [1]☐Positive [2]☐I don't know/he hasn't had a test [3]☐Negative [4]☐No regular partner

6. Which of the following best describes your usual frequency of HIV testing?

   [1]☐Every 3 months (or more) [2]☐Twice a year [3]☐Once a year [4]☐Less than once a year
   [5]☐I have never been tested before

7. Do you have any concerns regarding the accuracy or reliability of rapid HIV testing?

   [1]☐Yes [2]☐No [3]☐Don't know/Unsure

8. Would the availability of rapid HIV testing affect the frequency with which you have an HIV test?

   [1]☐Yes, I would test more often [2]☐No, I would not test more often [3]☐Don't know/Unsure

9. Where would you most prefer to have a **rapid** HIV test performed (if it was available)? (choose one)

   [1]☐Sexual health clinic [2]☐GP clinic [3]☐Community site e.g. ACON [4]☐Gay venue [5]☐Pharmacy
   [6]☐At home

10. Which of the following make it less likely for you to have a HIV test? (choose the most important reasons for you and leave those that do not apply to you blank)

    [1]☐I am scared of getting a HIV positive test result
    [2]☐I have been tested recently
    [3]☐I haven't done anything to put me at risk of HIV
    [4]☐I don't know where to go for a HIV test
    [5]☐It's difficult to find the time to get tested
    [6]☐It's difficult to get an appointment
    [7]☐It costs too much to get tested
    [8]☐I don't like showing my Medicare card when I get tested
    [9]☐I don't like having a discussion with the doctor/nurse/counsellor about getting tested
    [10]☐I don't like having blood taken for the test
    [11]☐I don't like needles/syringes
    [12]☐It's stressful waiting for the test result
    [13]☐It's annoying to have to return for the test result

11. Do you **currently** have sex with **casual** male partners?

    [1]☐Yes [2]☐No

12. Do you **currently** have sex with a **regular** male partner (or partners)?

    [1]☐Yes [2]☐No

13. How would you describe your sexual relationship with your current **regular** male partner? (choose one)

[1]☐I have **no** current regular male partner
[2]☐We are monogamous – **neither of us** has casual sex
[3]☐**Both my partner and I** have casual sex with other men
[4]☐I have casual sex with other men but **my partner doesn't**
[5]☐**My partner** has casual sex with other men but **I do not**
[6]☐I have **several regular** male partners

14. How many different men have you had sex with in the last 6 months?

[1]☐None [2]☐One [3]☐2-5 men [4]☐6-10 men [5]☐11-20 men [6]☐21-50 men [7]☐More than 50 men

15. In the last 6 months, how often did you use <u>condoms for anal sex</u> with **casual** male partners?

[1]☐Never [2]☐Sometimes [3]☐Always
[4]☐No anal sex with casual partners in the last 6 months
[5]☐No casual partners in the last 6 months

16. In the last 6 months, how often did you use <u>condoms for anal sex</u> with your **regular** male partner(s)?

[1]☐Never [2]☐Sometimes [3]☐Always
[4]☐No anal sex with regular partners in the last 6 months
[5]☐No regular partners in the last 6 months

PART TWO

17. Compared with the last time you had a HIV test, how long was your clinic visit today?

[1]☐Today's visit was shorter [2]☐It took about the same time [3]☐Today's visit was longer
[4]☐I haven't been tested for HIV before

18. How much **stress and anxiety** did you experience today having a rapid HIV test compared with the last time you had a conventional HIV test (before this visit)?

[1]☐**Much less** stress and anxiety than with conventional testing
[2]☐**Less** stress and anxiety than with conventional testing
[3]☐**Similar** stress and anxiety to conventional testing
[4]☐**More** stress and anxiety than with conventional testing
[5]☐**Much more** stress and anxiety than with conventional testing
[6]☐I haven't been tested for HIV before

19. Compared to having blood drawn from your arm today, how comfortable or uncomfortable was the **finger prick puncture** for the rapid HIV test?

   [1]☐The finger prick was much more uncomfortable
   [2]☐The finger prick was more uncomfortable
   [3]☐They were about the same
   [4]☐The finger prick was more comfortable
   [5]☐The finger prick was much more comfortable

20. I was satisfied with the **discussion** about rapid HIV testing **before** being tested

   [1]☐Strongly agree [2]☐Agree [3]☐Neutral [4]☐Disagree [5]☐Strongly disagree

21. I was satisfied with the **delivery** of my rapid HIV test **result**

   [1]☐Strongly agree [2]☐Agree [3]☐Neutral [4]☐Disagree [5]☐Strongly disagree

22. Overall, I was satisfied with the way the rapid HIV test was conducted by the staff at the clinic

   [1]☐Strongly agree [2]☐Agree [3]☐Neutral [4]☐Disagree [5]☐Strongly disagree

23. I would prefer to have a rapid HIV test rather than a conventional HIV test the **next time** (if available)

   [1]☐Strongly agree [2]☐Agree [3]☐Neutral [4]☐Disagree [5]☐Strongly disagree

24. I would **recommend** rapid HIV testing to someone else

   [1]☐Strongly agree [2]☐Agree [3]☐Neutral [4]☐Disagree [5]☐Strongly disagree

25. What is the most you would be prepared to **pay** for a rapid HIV test in a private clinic if it became available, but was not covered by Medicare? (choose one option only)

   [1]☐Nothing
   [2]☐$15
   [3]☐$20
   [4]☐$30
   [5]☐$50
